# A two-circular RNA signature as a noninvasive diagnostic biomarker for lung adenocarcinoma

**DOI:** 10.1186/s12967-019-1800-z

**Published:** 2019-02-18

**Authors:** Xiao-Xia Liu, Yi-E Yang, Xiao Liu, Meng-Yu Zhang, Rui Li, Yun-Hong Yin, Yi-Qing Qu

**Affiliations:** 1 Department of Respiratory and Critical Care Medicine, Qilu Hospital of Shandong University, Jinan, 250012 China; 2 Department of Clinical Laboratory, Qianfoshan Hospital of Shandong Province, Jinan, 250014 China

**Keywords:** LUAD, CircRNA, Plasma, Diagnosis, Biomarker, Hsa_circ_0005962, Hsa_circ_0086414

## Abstract

**Background:**

Recently, circular RNAs (circRNAs) have been reported to be microRNA sponges and play essential roles in cancer development. This study aimed to evaluate whether circulating circRNAs could be used as diagnostic biomarkers for lung adenocarcinoma (LUAD).

**Methods:**

The Gene Expression Omnibus (GEO) dataset was used to investigate differentially expressed circRNAs (DEcircRNAs) in paired LUAD tissues and adjacent nontumor tissues. The expression levels of the host genes were analyzed in The Cancer Genome Atlas (TCGA)-LUAD dataset, and the prognostic value was assessed using the Kaplan–Meier plotter. Quantitative real-time PCR (qRT-PCR) was performed to validate the expression of candidate circRNAs in the LUAD plasma and cells. The CCK8 assay was used to measure the function of circRNAs in cell proliferation. Competing endogenous RNA (ceRNA) network, gene ontology (GO) and Kyoto Encyclopedia of Genes and Genomes (KEGG) pathway analyses were performed to predict the possible mechanisms and functions of circRNAs in LUAD.

**Results:**

Two upregulated and two downregulated circRNAs were identified as candidate circRNAs using bioinformatics analysis. qRT-PCR demonstrated that hsa_circ_0005962 was upregulated in LUAD plasma and cells, whereas hsa_circ_0086414 was downregulated. Receiver operating characteristic (ROC) curve analysis confirmed that a signature comprising the two circRNAs had good diagnostic potential, with an area under the ROC curve (AUC) of 0.81 (*P* < 0.0001). In addition, we observed that overexpression of plasma hsa_circ_0086414 was related to EGFR mutations (*P* = 0.001). Plasma hsa_circ_0005962 displayed significantly different expression before and after surgery in patients with LUAD (*P* < 0.0001). In vitro experiments suggested that hsa_circ_0005962 promoted LUAD cell proliferation. For future studies, we predicted the circRNA-miRNA-mRNA network for hsa_circ_0005962. Bioinformatics analysis revealed that hsa_circ_0005962 might be involved in LUAD development.

**Conclusion:**

A circRNA signature was identified as a potential noninvasive biomarker for LUAD diagnosis.

**Electronic supplementary material:**

The online version of this article (10.1186/s12967-019-1800-z) contains supplementary material, which is available to authorized users.

## Background

Lung cancer is the leading cause of cancer-related death worldwide [1], and ~ 50% of these cancers are adenocarcinomas [2]. Despite improvements in diagnosis and treatment, the 5-year survival rate for lung cancer is approximately 18%, mainly because most patients are diagnosed at an advanced stage [3]. Therefore, discovering accurate and sensitive biomarkers is imperative and will be beneficial for the early diagnosis of lung adenocarcinoma (LUAD).

Circular RNAs (circRNAs), which have a circular covalently closed structure, are derived from precursor mRNA back-splicing of thousands of eukaryotic genes, endowing circRNAs with higher tolerance to exonuclease digestion [4]. Over the past few years, numerous potential functions of circRNAs have been discovered, such as acting as miRNA sponges, modulating transcription and interacting with RNA-binding proteins (RBPs) [5]. In addition, previous research showed that circRNAs were involved in autophagy, apoptosis, the cell cycle and proliferation, suggesting that circRNAs might have great significance for human disease [6]. Moreover, increasing studies have suggested that circRNAs are closely associated with different cancers, including lung cancer [7, 8], gastric cancer [9], colorectal cancer [10], hepatocellular carcinoma [11, 12] and breast cancer [13].

Recently, circRNAs have emerged as novel biomarkers due to their characteristics of abundance, stability, conservation, and specificity [6, 14, 15]. Moreover, circRNAs can steadily subsist not only in cancer tissues but also in exosomes and the blood [16, 17]. A liquid biopsy is more convenient and less invasive than traditional biopsy for analysis of biomarkers in tumor tissues. Hence, circulating circRNAs may be suitable for use as potential biomarkers for cancer diagnosis. Tan et al. [18] and Hang et al. [19] confirmed that plasma circRNAs might be potential biomarkers for non-small cell lung cancer (NSCLC) patients. However, little is known about the expression of plasma circRNAs in LUAD patients.

In the present study, we aimed to identify and validate potential plasma circRNA biomarkers for the diagnosis of LUAD. We performed bioinformatics analysis to select candidate LUAD-related circRNAs and validated the expression of these circRNAs in LUAD plasma and cells using quantitative real-time PCR (qRT-PCR). CircRNAs have been proposed to act as competing endogenous RNAs (ceRNAs) [6]. CeRNAs can function as miRNA sponges through their binding sites to modulate miRNA activity on target genes [5]. To predict the possible mechanisms and function of circRNAs in LUAD, a ceRNA network was constructed and a functional analysis was performed.

## Methods

### Selection of candidate circRNAs

CircRNA expression profiles for LUAD were searched in the Gene Expression Omnibus (GEO) database, and GSE101586 was selected. Normalized microarray data were re-analyzed using the GEO2R tool for comparison between LUAD tissues and paired nontumor tissues. The CircBase [20] database was used to find host genes related to the circRNAs, and the CSCD [21] database was used to select LUAD-specific circRNAs. Furthermore, the expression levels of their host genes were analyzed in The Cancer Genome Atlas (TCGA)-LUAD dataset downloaded from the Cancer Browser (https://xena.ucsc.edu/welcome-to-ucsc-xena/), and their prognostic value in LUAD was assessed using the Kaplan–Meier plotter [22].

### Patients and samples

Peripheral blood was collected from 153 LUAD patients at the Qilu Hospital of Shandong University between April and July 2018 for plasma isolation. The patients we assayed were in different TNM stages of LUAD, of which 83 were in stage I, 13 in stage II, 30 in stage III, and 25 in stage IV. The diagnosis of each case was confirmed through histological examination. None of the patients had a prior history of other cancers or metastatic cancer from other sites or had received chemotherapy or radiotherapy prior to plasma collection. Paired preoperative and postoperative blood samples (n = 54) were collected from the same patients before surgery and on the seventh day after resection. The 54 healthy controls without a history of any cancer were individually matched to the LUAD cases by age and gender. This study was approved by the Ethics Committee of Qilu Hospital of Shandong University (KYLL-2013-097; 25 February 2014), and written informed consent was obtained from all patients or their guardians.

### RNA isolation and reverse transcription

Total RNA was extracted from the patients’ plasma using the TRIzol™ LS Reagent (Invitrogen, Carlsbad, CA, USA) according to the manufacturer’s instructions. Whereas total RNA from the cells was isolated with the TRIzol Reagent (Invitrogen). The purity and concentration of the total RNA were evaluated with the NanoDrop Lite spectrophotometer (Thermo Scientific). The total RNA was subjected to cDNA synthesis using the PrimeScript™ RT Reagent Kit (Takara, Dalian, Liaoning, China). Briefly, 1000 ng of total RNA was reverse transcribed into cDNA with random primers in a final volume of 20 μL.

### qRT-PCR

The qRT-PCR was performed using the TB Green™ Premix Ex Taq™ II (TaKaRa) on the Applied Biosystems StepOnePlus Real-Time PCR System (Thermo Fisher Scientific). The PCR conditions were 95 °C for 30 s, followed by 40 cycles at 95 °C for 5 s and 60 °C for 30 s for each specific primer. Melting curves were generated at the end of amplification to ensure the specificity of the PCR products. Glyceraldehyde 3-phosphate dehydrogenase (GAPDH) was used as a reference gene, and the relative expression levels of the circRNAs were calculated using the 2^−ΔΔCT^ method. The divergent primers for these circRNAs were obtained from BioSune Corporation (Shanghai, China).

### Cell culture and transfection

All cell lines (A549, NCI-H1299, HCC827 and 16HBE) were purchased from Procell Life Science & Technology Co., Ltd. (Wuhan, China) and confirmed by short tandem repeat (STR) profiling. HCC827 is a LUAD cell line with an acquired mutation in the EGFR tyrosine kinase domain (E746-A750 deletion), and 16HBE is a human bronchial epithelial cell line. The A549, NCI-H1299 and HCC827 cells were cultured in RPMI-1640 medium (Gibco, Invitrogen, Carlsbad, CA), and the 16HBE cells were cultured in DMEM (Gibco) supplemented with 10% fetal bovine serum (FBS) and 1% penicillin/streptomycin. All cell lines were grown in humidified air at 37 °C with 5% CO_2_. The siRNA (si-hsa_circ_0005962, 5′-GAGACAACUUGACAUCUCUTT-3′) targeting the back-splice junction of hsa_circ_0005962 was synthesized by GenePharma (Shanghai, China). The A549 and H1299 cells were transfected with the siRNA using the Lipofectamine^®^ 2000 Reagent (Invitrogen) according to the manufacturer’s instructions. After transfection, the cells were processed to assess the knockdown activity by qRT-PCR or used for other experiments.

### Cell proliferation assay

To measure whether hsa_circ_0005962 was involved in cell proliferation, we performed the CCK8 assay. A549 and H1299 cells were seeded into 96-well plates at a density of 5 × 10^3^ cells per well after transfection and cultured for 24 h. Cell proliferation was assessed using the Cell Counting Kit-8 (CCK-8; Beyotime, Shanghai, China). The CCK-8 reagent was added to each well, and the cells were incubated at 37 °C for 2 h. The proliferation rates were determined at 0, 24, 48, 72, and 96 h. The optical density was measured by a microplate reader set at 450 nm. All experiments were repeated three times.

### CeRNA network analysis and function annotation

Potential interactions between the circRNAs and miRNAs were predicted using CircInteractome [23] based on the TargetScan algorithm. In addition, miRNA–target interactions were predicted with TargetScan [24] and miRTarBase [25]. The circRNA-miRNA-mRNA network was constructed and visualized with the Cytoscape [26] software. To gain further insight into the functions, gene ontology (GO) and Kyoto Encyclopedia of Genes and Genomes (KEGG) pathway analyses were performed for the target genes using DAVID v6.8 [27], The significant enrichment results were accepted at a threshold ≥ 2 gene counts with a *P* value < 0.05.

### Statistical analysis

All statistical data were analyzed using SPSS 22.0 (SPSS, Chicago, IL, USA), GraphPad 7.0 (GraphPad Software, San Diego, CA, USA) and the R software 3.5.1. The differences between the tumor and normal groups were evaluated using the nonparametric Mann–Whitney U test. A paired t test or Wilcoxon matched-pairs signed rank test was applied to compare differences in circRNA expression between the preoperative and postoperative groups. A Chi square test was used to analyze the associations between circRNA expression and clinicopathological factors in the LUAD patients. Logistic regression analysis was performed to establish a LUAD diagnostic panel consisting of two circRNAs. Receiver operating characteristic (ROC) curve analysis and the area under the ROC curve (AUC) were used to assess the diagnostic value of the circRNAs. The cutoff value of the circRNAs was calculated using the Youden index (specificity + sensitivity − 1). *P* values < 0.05 were considered statistically significant.

## Results

### Identification of candidate circRNAs

**Fig. 1:**
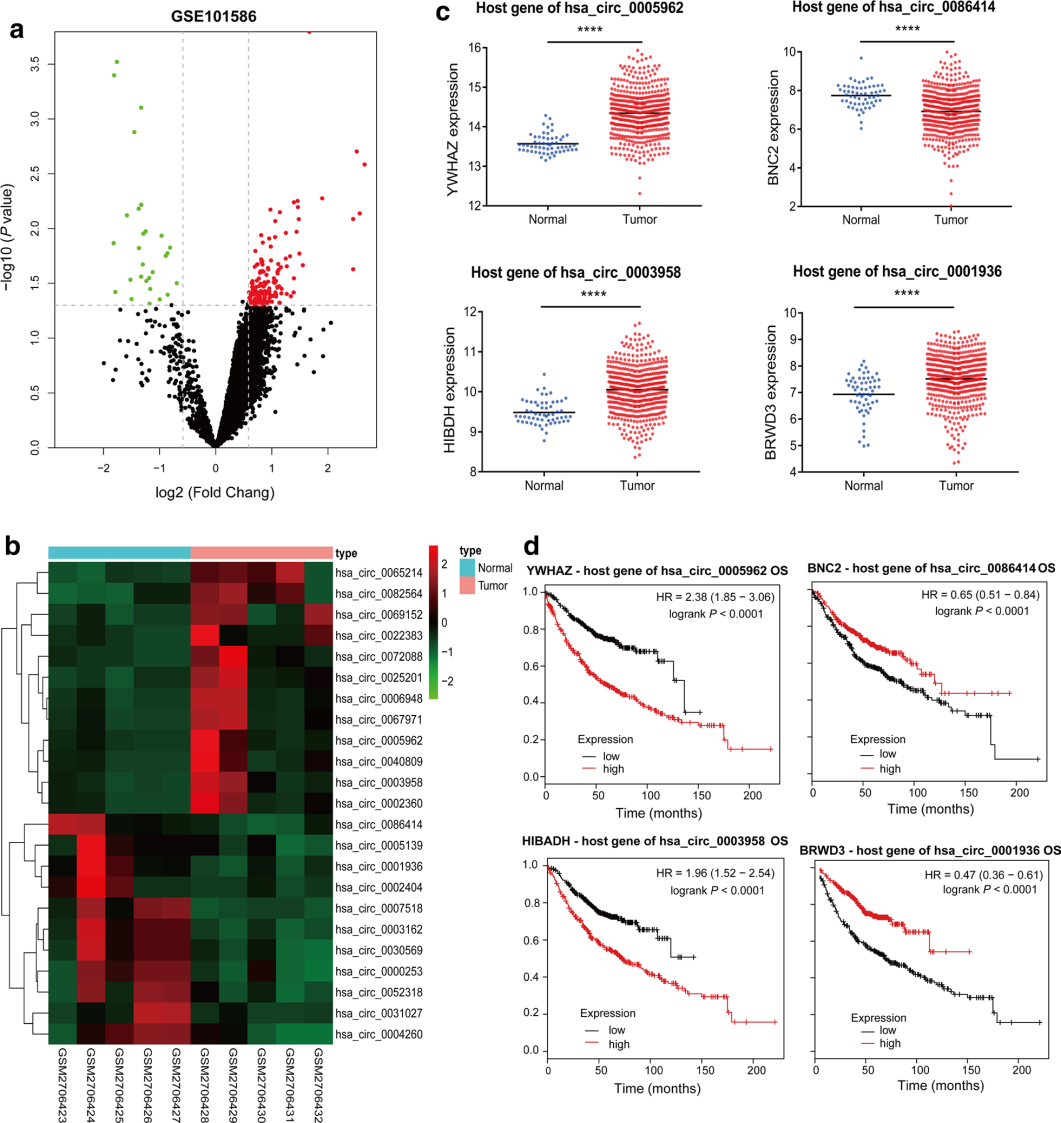
Circular RNA (circRNA) expression profiles of lung adenocarcinoma (LUAD) and matched nontumor tissues. **a** Volcano plot showing the differential expression of circRNAs between the tumor and normal groups. **b** Heatmap of the differential expression of the 23 selected LUAD-specific circRNAs in the GSE101586 dataset. **c** The expression of host genes of hsa_circ_0005962, hsa_circ_0003958, hsa_circ_0086414 and hsa_circ_0001936 in The Cancer Genome Atlas (TCGA)-LUAD dataset. **d** Overall survival analysis for the host genes of the four circRNAs in LUAD patients was performed using the Kaplan–Meier plotter. Log-rank tests were used to determine statistical significance. ****P < 0.0001

In the present study, we re-analyzed the microarray GSE101586 data [7] from the GEO database and detected differentially expressed circRNAs (DEcircRNAs) in paired LUAD tissues and adjacent nontumor tissues using the GEO2R tool. A total of 182 DEcircRNAs (*P* < 0.05 and fold change > 1.5) were obtained. Among the 182 deregulated circRNAs, 28 were upregulated and 79 were downregulated (Fig. 1a). Next, we utilized the CircBase and CSCD databases to select LUAD-specific circRNAs. As a result, 23 LUAD-specific circRNAs were eventually detected. The differential expression of the 23 selected LUAD-specific circRNAs in the GSE101586 dataset is shown in Fig. 1b. Next, to identify candidate circRNAs, we analyzed the expression levels of their host genes in TCGA lung samples and the prognostic value of their host genes using the Kaplan–Meier plotter. Finally, two upregulated (hsa_circ_0005962, hsa_circ_0003958) and two downregulated circRNAs (hsa_circ_0086414, hsa_circ_0001936) were identified as candidate circRNAs, because their host genes showed differential expression in the LUAD and adjacent normal tissues. We found that these circRNAs had prognostic value. The differential expression and survival analyses of the host genes of the four candidate circRNAs are shown in Fig. 1c, d, respectively.

### Validating the expression of the candidate circRNAs

**Fig. 2:**
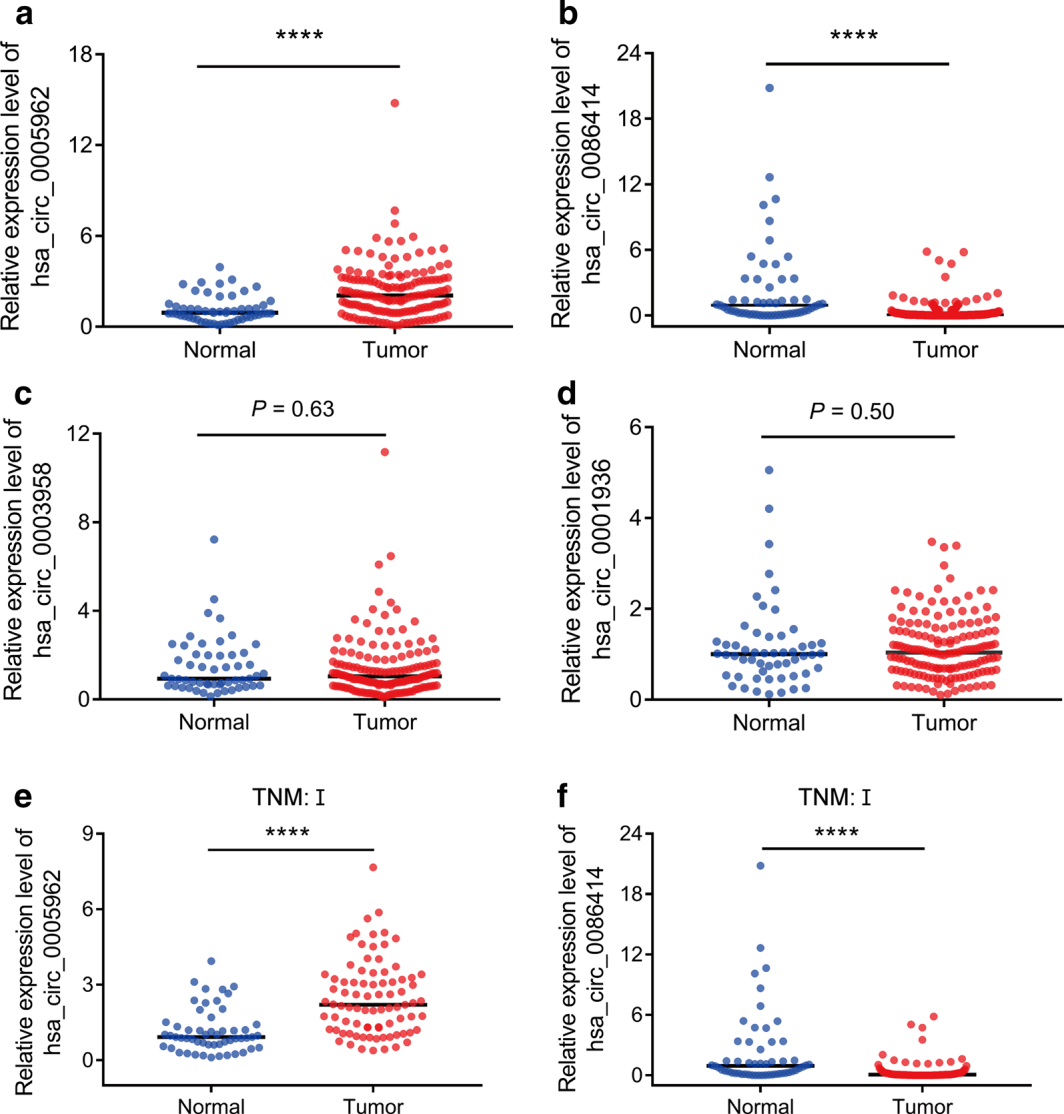
Quantitative real-time PCR (qRT-PCR) analysis of the candidate circRNAs in the LUAD patients and normal controls. **a** Hsa_circ_0005962 expression was upregulated in the plasma of the LUAD patients compared with that of the normal controls. **b** Hsa_circ_0086414 was downregulated in the LUAD patients. Hsa_circ_0003958 (**c**) and hsa_circ_0001936 (**d**) were detected without significantly differential expression between the two groups. **e** Hsa_circ_0005962 was upregulated in LUAD patients with TNM stage I. **f** Hsa_circ_0086414 was downregulated in LUAD patients with TNM stage I. ****P < 0.0001

The expression of the four candidate circRNAs was verified in 153 primary LUAD and 54 normal plasma samples using qRT-PCR. The results showed that hsa_circ_0005962 was highly upregulated in LUAD (*P* < 0.0001, Fig. 2a) and hsa_circ_0086414 was downregulated (*P* < 0.0001, Fig. 2b), which was consistent with the microarray analysis results. However, hsa_circ_0003958 and hsa_circ_0001936 showed no significantly differential expression in the plasma between the LUAD patients and the normal controls (*P* = 0.63, Fig. 2c; *P* = 0.50, Fig. 2d). Furthermore, we found that hsa_circ_0005962 (*P* < 0.0001, Fig. 2e) and hsa_circ_0086414 (*P* < 0.0001, Fig. 2f) were differentially expressed in early LUAD patients and normal controls.

### Diagnostic value of hsa_circ_0005962 and hsa_circ_0086414 for LUAD patients

**Fig. 3:**
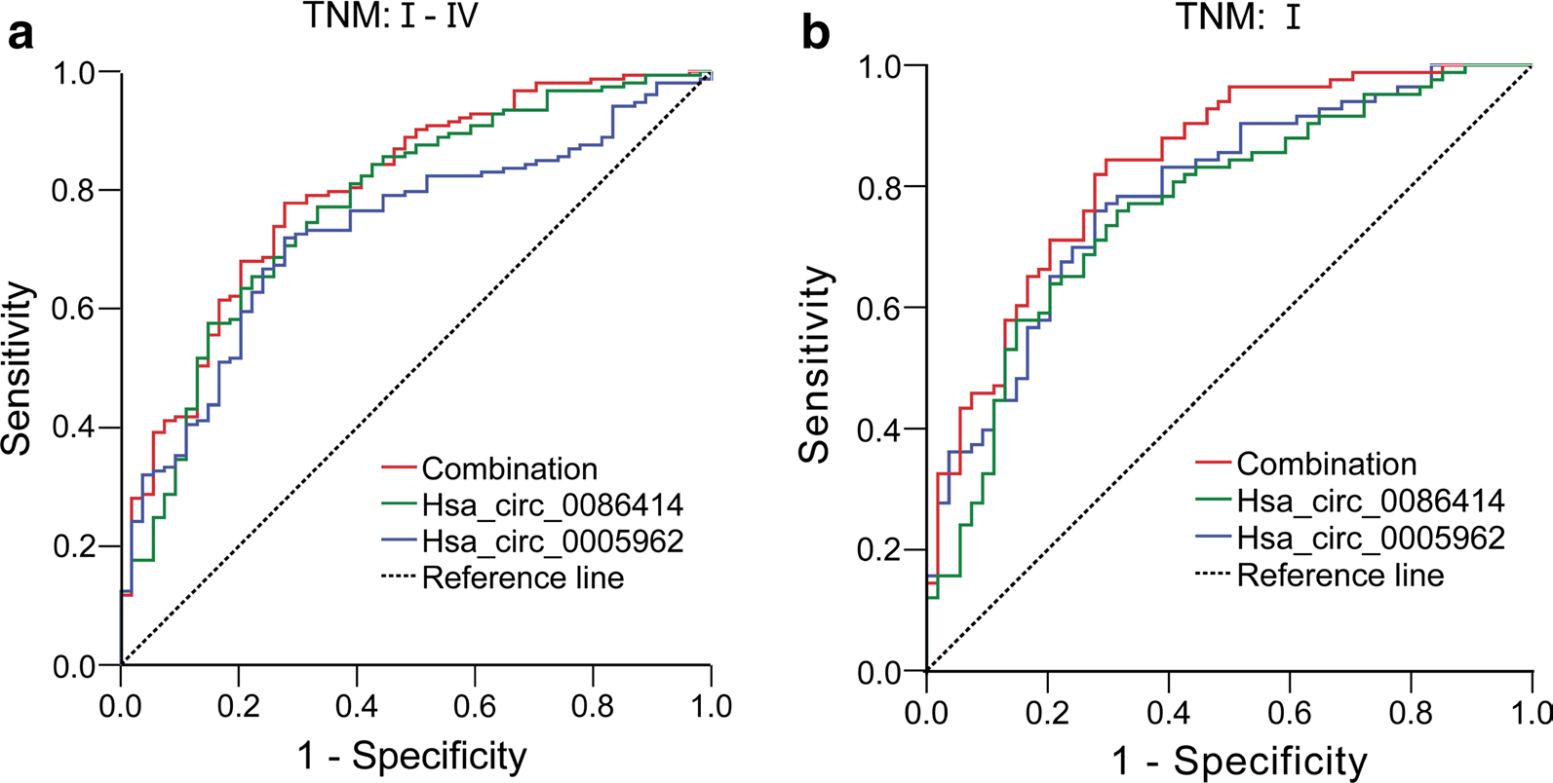
ROC analysis of hsa_circ_0005962, hsa_circ_0086414 and the combination of the two markers for the diagnosis of LUAD. **a** TNM stages I-IV; Combination: The area under the ROC curve (AUC) 0.81, *P* < 0.0001; Hsa_circ_0005962: AUC 0.73, *P* < 0.0001; Hsa_circ_0086414: AUC 0.78, *P* < 0.0001. **b** TNM stage I; Combination: AUC 0.83, *P* < 0.0001; Hsa_circ_0005962: AUC 0.79, *P* < 0.0001; Hsa_circ_0086414: AUC 0.77, *P* < 0.0001

ROC curve analysis was used to investigate the diagnostic value of hsa_circ_0005962 and hsa_circ_0086414 in distinguishing LUAD patients from normal controls. As shown in Fig. 3a, the AUC of hsa_circ_0005962 was 0.73 and the optimal cut-off value was 1.21, with a sensitivity of 71.90% and specificity of 72.22% (*P* < 0.0001). For hsa_circ_0086414, the AUC was 0.78 and the cut-off value was 0.39, with a sensitivity and specificity of 77.12% and 66.67%, respectively (*P* < 0.0001). Notably, the combination of the two circRNA expression values provided the best discrimination, with an AUC of 0.81, sensitivity of 77.80% and specificity of 72.22% (*P* < 0.0001). Furthermore, we analyzed the diagnostic performance of the two-circRNA signature in distinguishing LUAD patients with early TNM stage from healthy individuals. The AUC of the signature for LUAD patients with TNM stages I was 0.83 (*P* < 0.0001, Fig. 3b).

### Correlation between expression of the two circRNAs and the clinicopathological characteristics

An additional analysis was performed to assess correlations between the expression levels of the two circRNAs and the clinicopathological features of the LUAD patients. As indicated in Table 1, hsa_circ_0005962 and hsa_circ_0086414 expression was associated with gender (*P* = 0.024; *P* = 0.046). Furthermore, a high plasma hsa_circ_0086414 expression level was correlated with EGFR mutations (*P* = 0.001). However, we did not observe any association between circRNAs and the patients’ ages, smoking history, tumor sizes, lymphatic metastasis, metastasis, CEA levels or TNM stages.

**Table 1:** Association between the plasma circRNA expression levels and clinicopathological characteristics of LUAD patients

Characteristics	n = 153	Hsa_circ_0005962			Hsa_circ_0086414		
		Low (n = 43)	High (n = 110)	*P* value	Low (n = 117)	High (n = 36)	*P* value
*Age (years)*							
< 60	74	22	52	0.665	58	16	0.59
≥ 60	79	21	58		59	20	
*Gender*							
Female	93	20	73	*0.024*	66	27	*0.046*
Male	60	23	37		51	9	
*Smoking*							
No	99	23	76	0.097	72	27	0.089
Yes	53	19	34		45	8	
*Tumor size*							
≤ 3 cm	99	25	74	0.907	75	24	0.956
> 3 cm	42	11	31		32	10	
*Lymphatic metastasis*							
Negative	111	27	84	0.091	87	24	0.366
Positive	42	16	26		30	12	
*Metastasis*							
M0	130	33	97	0.075	99	31	0.826
M1	23	10	13		18	5	
*TNM stage*							
I	83	20	63	0.282	64	19	0.728
II and III and IV	68	21	47		51	17	
*CEA*							
Negative	75	16	59	0.146	56	19	0.337
Positive	45	15	30		37	8	
*EGFR mutation*							
Negative	26	10	16	0.287	25	1	*0.001*
Positive	42	11	31		25	17	

### Plasma hsa_circ_0005962 showed differential expression in LUAD patients before and after surgical resection

**Fig. 4:**
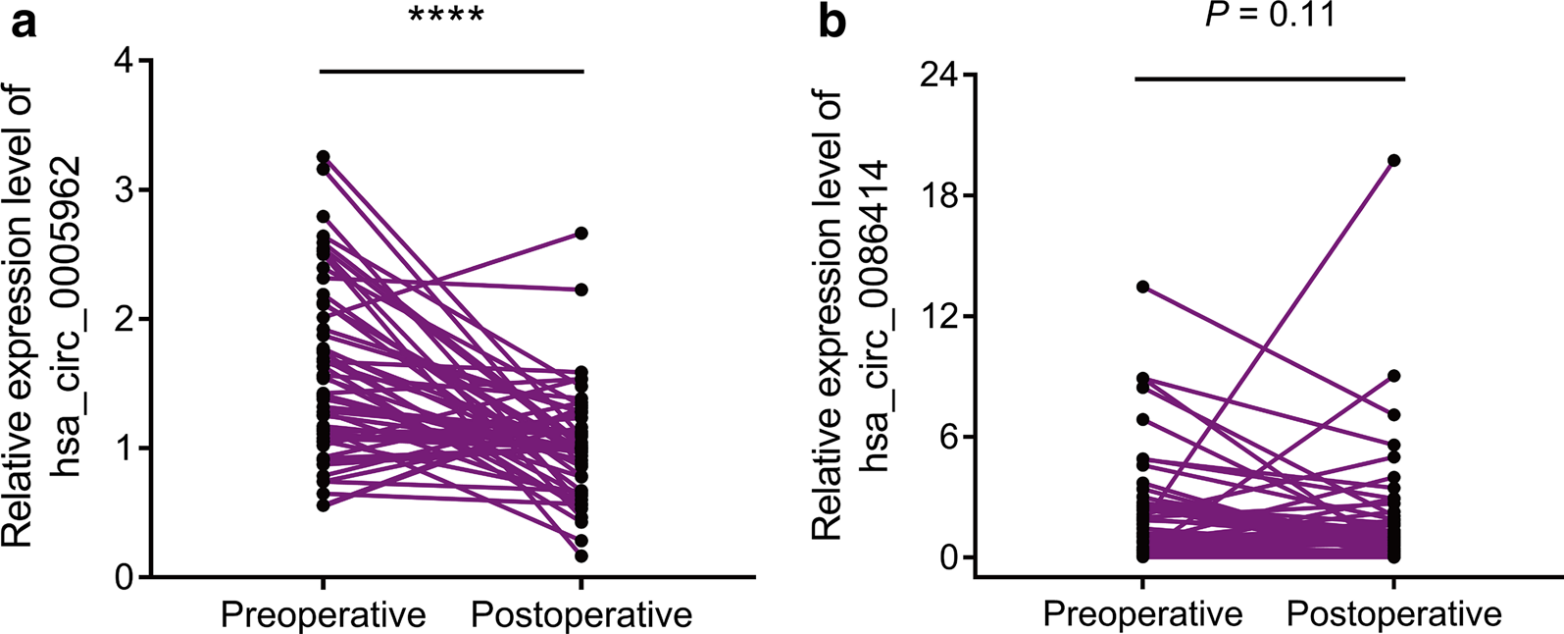
Comparison of plasma hsa_circ_0005962 and hsa_circ_0086414 expression in preoperative and postoperative LUAD patients. **a** Upregulated hsa_circ_0005962 was detected at a lower level in the plasma samples after surgery. **b** No significant differential expression of hsa_circ_0086414 was detected in the patients before and after surgery. ****P < 0.0001

Next, we examined hsa_circ_0005962 and hsa_circ_0086414 expression in the plasma of LUAD patients before and after surgery. The results indicated that the hsa_circ_0005962 expression level was decreased in 42 of the 54 (77.78%) LUAD patients after surgery (*P* < 0.0001, Fig. 4a). However, no significant difference in hsa_circ_0086414 expression was observed between the preoperative and postoperative stages (*P* = 0.11, Fig. 4b).

### Hsa_circ_0005962 promotes the proliferation of LUAD cell lines in vitro

**Fig. 5:**
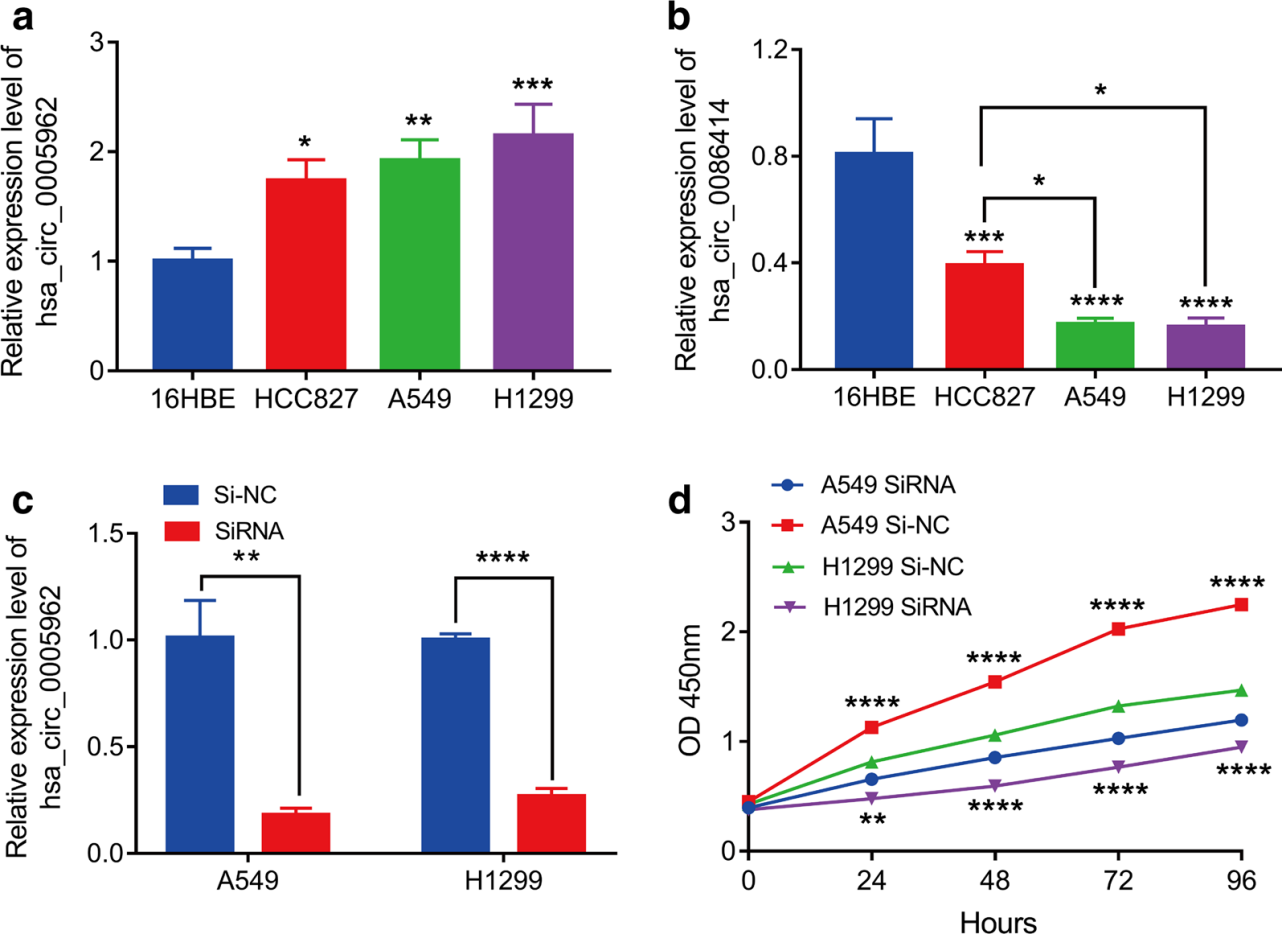
Hsa_circ_0005962 promotes LUAD cell proliferation in vitro. **a** Hsa_circ_0005962 expression was upregulated in the LUAD cells (A549, H1299 and HCC827) compared to that of the normal human bronchial epithelial cells (16HBE). **b** Hsa_circ_0086414 was downregulated in the LUAD cells compared to that of the 16HBE cells and was differentially expressed between the LUAD cells with (HCC827) and without (A549 and H1299) EGFR mutations. **c** qRT-PCR analysis of hsa_circ_0005962 expression in A549 and H1299 cells after transfection with a specifically synthesized siRNA. **d** The CCK-8 assay showed that hsa_circ_0005962 promoted A549 and H1299 cell proliferation. *P < 0.05; **P < 0.01; ***P < 0.001; ****P < 0.0001

To further explore the role of circRNAs in LUAD, we performed a preliminary in vitro experiment. First, we examined hsa_circ_0005962 and hsa_circ_0086414 expression in LUAD cell lines. The results showed that hsa_circ_0005962 was upregulated in the LUAD cell lines compared with that in human bronchial epithelial cells (Fig. 5a), whereas hsa_circ_0086414 was downregulated (Fig. 5b). Because hsa_circ_0005962 was significantly upregulated in LUAD plasma and cells, it was used as a target to investigate the role of circRNAs in LUAD tumorigenesis. A549 and H1299 cells were transfected with si-hsa_circ_0005962 or a negative control siRNA (si-NC). The qRT-PCR revealed that hsa_circ_0005962 expression was downregulated in LUAD cells by the siRNA compared with that of the cells treated with the si-NC (Fig. 5c). To explore whether hsa_circ_0005962 was involved in cell proliferation, we performed the CCK8 assay. We determined that knockdown of hsa_circ_0005962 greatly suppressed A549 and H1299 cell proliferation (Fig. 5d). These in vitro experiments suggested that hsa_circ_0005962 promoted LUAD cell proliferation.

### Prediction of the ceRNA network for hsa_circ_0005962

**Fig. 6:**
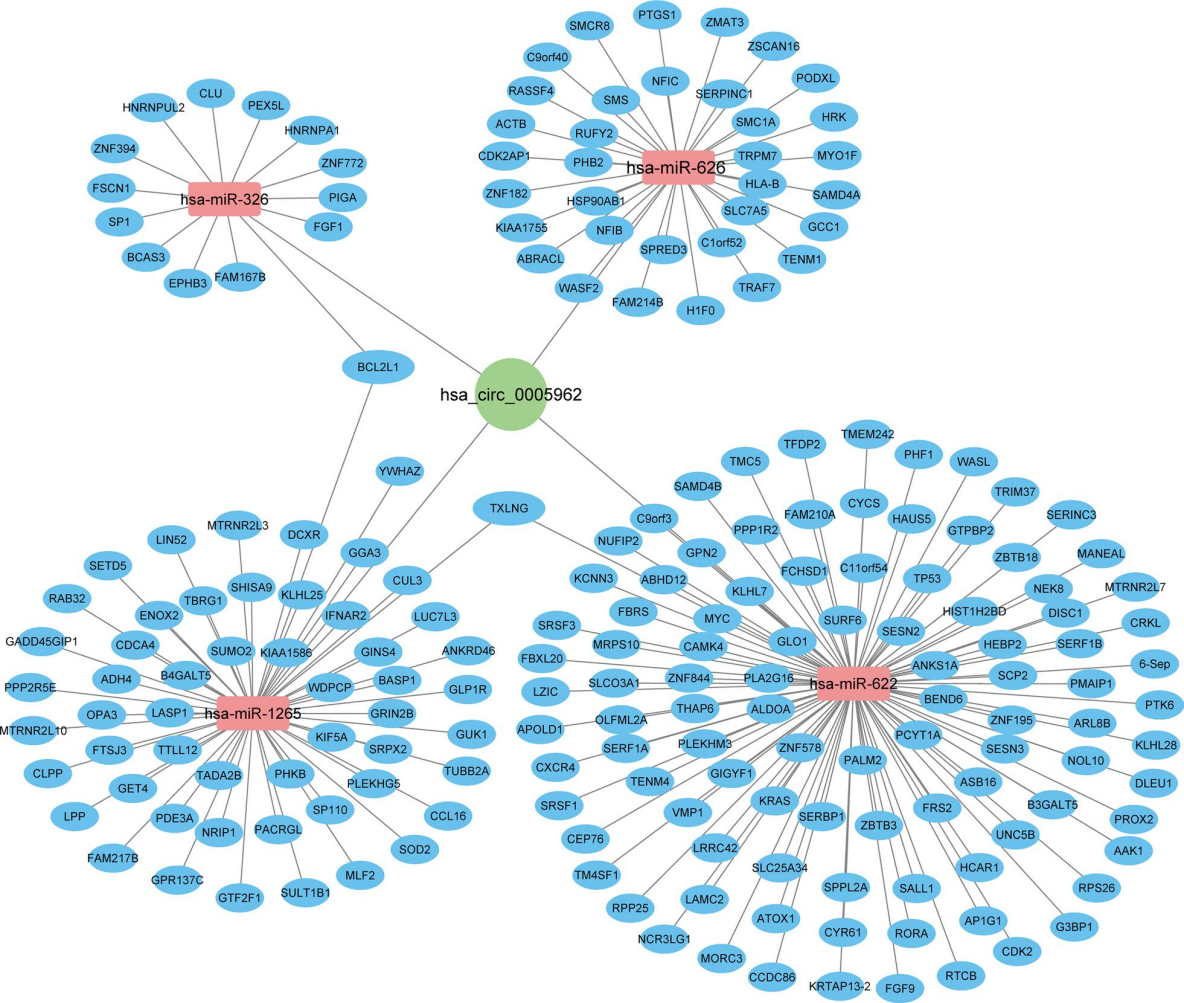
Bioinformatics analysis of the hsa_circ_0005962–miRNA–mRNA interactions. The competing endogenous RNA (ceRNA) network was constructed and visualized with the Cytoscape software

Based on the CircInteractome prediction, the top six miRNAs (hsa-miR-369-5p, hsa-miR-626, hsa-miR-326, hsa-miR-330-5p, hsa-miR-1265, and hsa-miR-622) targeted by hsa_circ_0005962 were identified. Then, we predicted the target mRNAs of these miRNAs in the TargetScan and miRTarBase databases. The 4 miRNAs and 203 target mRNAs are illustrated in Fig. 6. We predict that hsa-miR-1265 may directly target YWHAZ, which is the host gene of hsa_circ_0005962. Moreover, YWHAZ expression was increased in the LUAD compared with the normal control tissues in TCGA dataset (Fig. 1c, *P* < 0.0001). The microarray data from the Kaplan–Meier plotter indicated that patients with high YWHAZ expression levels had significantly poorer overall survival (Fig. 1d, *P* < 0.0001, HR = 2.38, 95% CI 1.85–3.06).

### Functional enrichment analysis of the hsa_circ_0005962 target genes

**Fig. 7:**
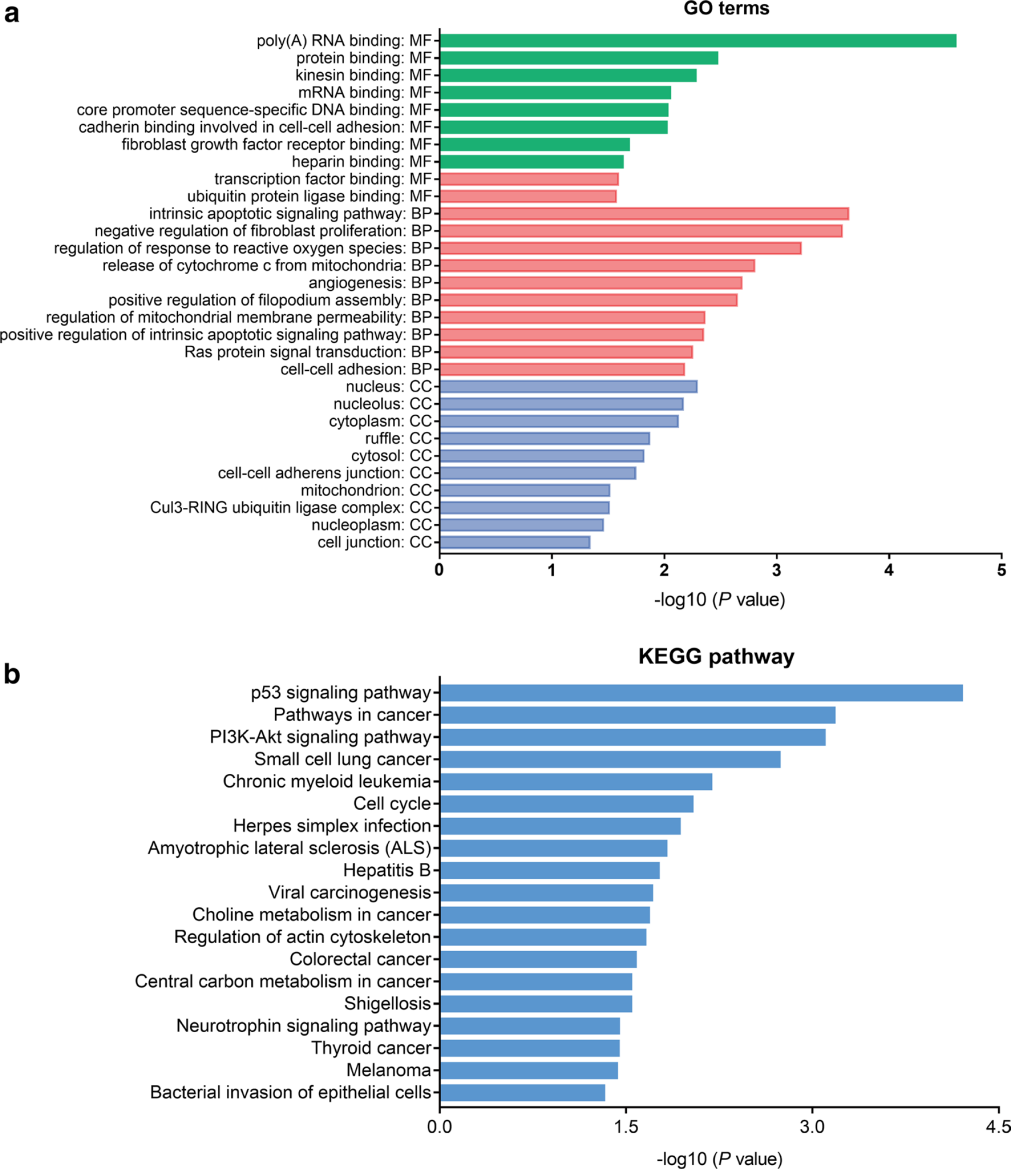
Functional enrichment analysis for hsa_circ_0005962. **a** Gene ontology (GO) analysis of hsa_circ_0005962 based on the ceRNA network. The top 10 significantly enriched biological processes (BPs), cellular components (CCs) and molecular functions (MFs) are listed. **b** Kyoto Encyclopedia of Genes and Genomes (KEGG) pathway analysis of hsa_circ_0005962 based on the ceRNA network

Finally, GO analysis was conducted for hsa_circ_0005962. The top 10 significantly enriched biological processes (BPs), cellular components (CCs) and molecular functions (MFs) are shown in Fig. 7a. The main annotations included poly(A) RNA binding, protein binding, DNA binding and regulation of transcription and cell adhesion. Based on KEGG annotation, 18 pathways were identified, many of which were cancer-related, such as the p53 signaling pathway, pathways in cancer, PI3K-Akt signaling pathway, small cell lung cancer, chronic myeloid leukemia and cell cycle (Fig. 7b).

## Discussion

Early diagnosis has great significance for the treatment and prognosis of LUAD. Unlike linear RNAs, circRNAs are expected to be novel candidates for biomarker detection, since they are more abundant and stable in body fluids (including serum exosomes, plasma and saliva) [16, 28, 29]. Recently, an increasing number of studies has noted that circRNAs can be used as biomarkers for cancer diagnosis [6]. Hence, in this study, we aimed to identify circulating circRNAs that could be used as biomarkers for the diagnosis of LUAD.

CircRNAs are derived from linear RNAs, most of which are produced by back splicing of exons [30]. A previous study demonstrated that most circRNAs were associated with their linear RNA expression during tumorigenesis [31]. In this study, we used a GEO dataset to investigate DEcircRNAs in LUAD and then selected the DEcircRNAs whose host genes were differentially expressed in TCGA-LUAD data and associated with the prognosis as candidate circRNAs. We speculated that these candidate DEcircRNAs might be involved in LUAD pathogenesis.

Furthermore, we validated the expression of the candidate circRNAs in the LUAD patient plasma. To the best of our knowledge, this report is the first on hsa_circ_0005962 and hsa_circ_0086414 expression in the plasma of patients with cancer. Our study utilized plasma samples due to their advantages of availability and noninvasiveness. Zhu et al. [32] found differential expression of plasma hsa_circ_0013958 between LUAD patients and healthy controls. However, their sample size was relatively small (30 LUAD cases and 30 healthy controls). In the present study, we verified the plasma circRNA expression levels in a relatively larger sample set (153 LUAD and 54 normal samples). We found that both hsa_circ_0005962 and hsa_circ_0086414 were differentially expressed in the plasma of LUAD patients and that the combination of these two molecules improved the diagnostic accuracy for LUAD. In addition, we found that hsa_circ_0005962 and hsa_circ_0086414 were differentially dysregulated in the early stage of LUAD, suggesting that they may be the promising diagnostic biomarkers for the early stage of LUAD. Although the other two candidate circRNAs showed no significantly differential expression in the plasma, we attributed this discrepancy to differences in the testing methods (microarray analysis vs qRT-PCR), sample types (tissue vs plasma) and sample sizes. The above results suggested that the two-circRNA signature could be used as a potential noninvasive biomarker for diagnosis of LUAD.

Zhou et al. [31] detected a large number of circRNAs in plasma of cervical cancer patients showing differential expression before and after surgery, and some of these circRNAs were indicated as prognostic markers, suggesting that these plasma circRNAs may be associated with cancer progression. Li et al. [33] confirmed that the changes in plasma hsa_circ_0001017 and hsa_circ_0061276 expression before and after surgery were independent monitoring indicators for gastric cancer recurrence. In this study, we observed that hsa_circ_0005962 expression was decreased in postoperative LUAD patients compared to that in the preoperative patients, suggesting that it may be associated with the progression of LUAD. This decrease may be due to the decreasing release of tumor-derived nucleic acids after tumor resection [34], resulting in significant changes in the plasma hsa_circ_0005962 levels before and after surgery. However, no significant difference in hsa_circ_0086414 expression was found between the preoperative and postoperative stages. It had been reported that the co-precipitation of circRNA with exosomes might be a possible mechanism for circRNA clearance [35]. Therefore, we may attribute this result to the increasing clearance of circRNA through exosomes, so that the expression of hsa_circ_0086414 was not significantly increased after surgery.

EGFR, which is the epidermal growth factor receptor, is a member of the ERBB receptor tyrosine kinase family that promotes cell survival, proliferation and invasion [36]. Mutations in EGFR are important drivers of NSCLC, and EGFR-targeted therapy can effectively improve the prognosis of patients with advanced NSCLC [37]. EGFR mutations occur mainly in adenocarcinoma, younger women, and never-smokers [38]. Surprisingly, we found that hsa_circ_0086414 was highly expressed in EFGR mutant patients compared to EGFR wild-type patients (*P* < 0.01, Additional file 1. Figure S1A), and was more highly expressed in female patients than male patients (*P* < 0.05, Additional file 1. Figure S1B). Previous studies showed that miRNAs could be involved in the development of EGFR mutations in LUAD [39–41]. Therefore, we hypothesized that hsa_circ_0086414 might contribute to EGFR mutation by binding miRNAs. To explore the possible mechanism, bioinformatics analysis identified 9 miRNAs might be the targets of hsa_circ_0086414, which were differentially expressed in patients with and without EGFR mutations (Additional file 2). Among the 9 miRNAs, hsa-miR-103a-3p was reported to inhibit the EGFR expression via EGFR/KRAS pathway [42]. However, the exact mechanism should be performed in the future study.

CircRNAs have been reported to regulate mRNA expression by competing for miRNAs [4]. For instance, circRNA ciRS-7 [43] and CDR1as [44] can bind to miR-7 and function as miRNA sponges. In this study, the CCK8 assay suggested that hsa_circ_0005962 might promote cell proliferation in LUAD. To further study this possibility, we predicted the hsa_circ_0005962-miRNA-target network and performed a functional enrichment analysis. As a result, 4 miRNAs (hsa-miR-626, hsa-miR-326, hsa-miR-1265, and hsa-miR-622) and their 203 target genes were identified. Previous studies showed that hsa-miR-326 inhibited SMO expression in glioma cancer and CD34(+) chronic myeloid leukemia cells and acted as a tumor suppressor miRNA by inhibiting the PI3 kinase pathway in glioblastomas [45–47]. In addition, hsa-miR-326 was reported to regulate lung cancer metastasis and invasion [48, 49]. Research confirmed that hsa-miR-622 was downregulated in hepatocellular carcinoma, resulting in dysregulation of CXCR4 and KRAS [50, 51]. Interestingly, YWHAZ, which is the host gene of hsa_circ_0005962, was predicted to be the target of hsa-miR-1265. Moreover, bioinformatics analysis showed that YWHAZ was upregulated in LUAD and associated with the prognosis. Previous research certified that YWHAZ (also known as 14-3-3zeta) was overexpressed in NSCLC and promoted cancer progression [52, 53]. These findings supported the hypothesis that hsa_circ_0005962 might function as a sponge for hsa-miR-1265, thus increasing expression levels of YWHAZ and promoting tumorigenesis of LUAD. Functional enrichment analysis revealed that its target genes were involved in several cancer-related pathways, including the p53 signaling pathway, pathways in cancer, PI3K-Akt signaling pathway, small cell lung cancer, chronic myeloid leukemia and cell cycle. This evidence indicated that hsa_circ_0005962 might act as a miRNA sponge to promote the development of LUAD and further research on its mechanism is worthwhile.

Finally, to better understand the clinical application of circRNAs, we searched the clinical trials using the term “circRNAs” from the website https://clinicaltrials.gov/, and https://www.who.int/ictrp/en/. Interestingly, there were recruiting clinical trial for circRNAs as biomarkers for prostate cancer (ChiCTR1800019529), acute myocardial infarction (ChiCTR1800019218) and acute lung injury (NCT03766204) in the year 2018. However, there were no ongoing clinical trials for circRNAs biomarker for lung cancer. Our present study provided the evidence for circulating circRNAs as noninvasive biomarkers for lung cancer.

## Conclusions

In conclusion, the present study validated the significant upregulation of hsa_circ_0005962 and downregulation of hsa_circ_0086414 in LUAD plasma and cells, suggesting the potential for use of this two-circRNA signature as a novel noninvasive biomarker for LUAD diagnosis. Furthermore, we found that the hsa_circ_0086414 levels were associated with EGFR mutations. Hsa_circ_0005962 had different expression in the plasma of LUAD patients before and after surgery, and in vitro experiments and in silico analysis indicated that it might be involved in the development of LUAD.

## Additional files

**Additional file 1: Figure S1.** Correlation between hsa_circ_0086414 expression and EGFR mutations and gender. (**A**) Hsa_circ_0086414 was highly expressed in EGFR mutant patients compared to EGFR wild-type patients. (**B**) Hsa_circ_0086414 was more highly expressed in female patients than male patients. *P < 0.05; **P < 0.01.

**Additional file 2: Table S1.** The predicted miRNA targets for hsa_circ_0086414 in the CircBank database. **Table S2.** The miRNAs that were significantly differentially expressed between the EGFR mutated and EGFR wild-type LUAD patients in the GSE119268 dataset. **Table S3.** Nine miRNAs might be the targets for hsa-circ-0086414 and that were differentially expressed in patients with and without EGFR mutations.

## Abbreviations

circRNAs: circular RNAs
LUAD: lung adenocarcinoma
GEO: Gene Expression Omnibus
DEcircRNAs: differentially expressed circRNAs
TCGA: The Cancer Genome Atlas
qRT-PCR: quantitative real-time PCR
ceRNA: competing endogenous RNA
GO: gene ontology
KEGG: Kyoto Encyclopedia of Genes and Genomes
ROC: receiver operating characteristic
AUC: area under the ROC curve
RBPs: RNA-binding proteins
NSCLC: non-small cell lung cancer
GAPDH: glyceraldehyde 3-phosphate dehydrogenase
BP: biological process
CC: cellular component
MF: molecular function
