# Supplementary figures and images for: A two-circular RNA signature as a noninvasive diagnostic biomarker for lung adenocarcinoma
Source: J Transl Med. 2019 Feb 18;17:50. doi: 10.1186/s12967-019-1800-z (PMC6380039; doi:10.1186/s12967-019-1800-z)

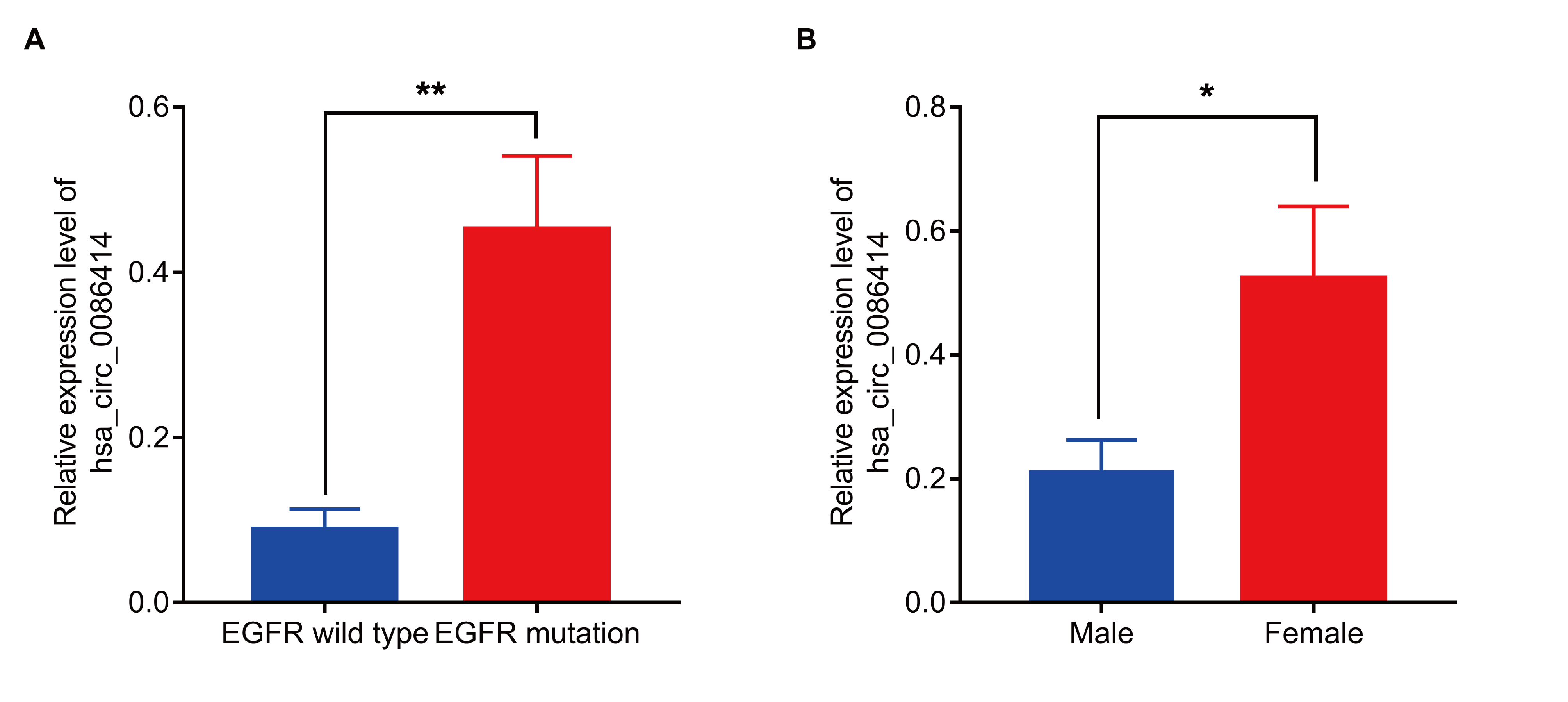

Supplement: Supplementary file 1 — Additional file 1: Figure S1. Correlation between hsa_circ_0086414 expression and EGFR mutations and gender. (A) Hsa_circ_0086414 was highly expressed in EGFR mutant patients compared to EGFR wild-type patients. (B) Hsa_circ_0086414 was more highly expressed in female patients than male patients. *P < 0.05; **P < 0.01. [file 12967_2019_1800_MOESM1_ESM.tif]
